# Diagnosis codes underestimate chronic kidney disease incidence compared with eGFR-based evidence: a retrospective observational study of patients with type 2 diabetes in UK primary care

**DOI:** 10.3399/BJGPO.2023.0079

**Published:** 2024-01-24

**Authors:** Rose Sisk, Rory Cameron, Waqas Tahir, Camilla Sammut-Powell

**Affiliations:** 1 Gendius Ltd, Alderley Edge, UK; 2 Affinity Care, National Health Service, Bradford, UK

**Keywords:** diagnosis, diabetes, renal medicine, renal insufficiency, chronic, primary health care

## Abstract

**Background:**

Type two diabetes (T2D) is a leading cause of both chronic kidney disease (CKD) and onward progression to end-stage renal disease. Timely diagnosis coding of CKD in patients with T2D could lead to improvements in quality of care and patient outcomes.

**Aim:**

To assess the consistency between estimated glomerular filtration rate (eGFR)-based evidence of CKD and CKD diagnosis coding in UK primary care.

**Design & setting:**

A retrospective analysis of electronic health record data in a cohort of people with T2D from 60 primary care centres within England between 2012 and 2022.

**Method:**

We estimated the incidence rate of CKD per 100 person–years using eGFR-based CKD and diagnosis codes. Logistic regression was applied to establish which attributes were associated with diagnosis coding. Time from eGFR-based CKD to entry of a diagnosis code was summarised using the median and interquartile range.

**Results:**

The overall incidence of CKD was 2.32 (95% confidence interval [CI] = 2.24 to 2.41) and significantly higher for eGFR-based criteria than diagnosis codes: 1.98 (95% CI = 1.90 to 2.05) versus 1.06 (95% CI = 1.00 to 1.11), respectively; *P*<0.001. Only 45.4% of CKD incidences identified using eGFR-based criteria had a corresponding diagnosis code. Patients who were younger, had a higher CKD stage (G4), had an observed urine albumin-to-creatinine ratio (A1), or no observed HbA1c in the past year were more likely to have a diagnosis code.

**Conclusion:**

Diagnosis coding of patients with eGFR-based evidence of CKD in UK primary care is poor within patients with T2D, despite CKD being a well-known complication of diabetes.

## How this fits in

Type two diabetes (T2D) is a recognised cause of chronic kidney disease (CKD), and early identification and management of CKD can reduce the risk of progression and related complications. Diagnosis coding of CKD is associated with better patient outcomes, yet we have observed that less than half of patients with T2D who meet eGFR-based criteria for stage 3–5 CKD have a CKD diagnosis code in their primary care record. There is a need to understand why CKD diagnosis coding practices are subpar in primary care and this research acts as a call to action to improve.

## Introduction

Chronic kidney disease (CKD) is a progressive condition that places an enormous burden on healthcare systems and patients globally.^1^ Type two diabetes (T2D) is a leading cause of CKD and is associated with rapid progression and an increased risk of end-stage renal disease,^2,3^ and both CKD and T2D are risk factors for cardiovascular complications.^4^ Regular kidney function testing in people with T2D therefore facilitates early detection and appropriate management of CKD.^5,6^

CKD is characterised by a sustained drop in glomerular filtration rate, often evidenced by a reduced estimated glomerular filtration rate (eGFR), and/or persistent albuminuria.^7^ The combination of eGFR, calculated using serum creatinine, and albuminuria, with severity characterised using the urine albumin-to-creatinine ratio (UACR), are used to classify CKD and estimate the risk of progression.^7^ Existing clinical practice guidelines therefore dictate that people with T2D should receive serum creatinine and urinary albumin tests annually.^8,9^ In the UK, these tests take place during annual reviews of patients with T2D in primary care; however, albumin testing uptake remains extremely poor compared with serum creatinine testing.^10^

Early identification of CKD within primary care can facilitate earlier intervention to slow down its progression and reduce the risk of complications. Such interventions may include lifestyle advice and targeted risk factor management via pharmaceutical intervention,^11,12^ including sodium glucose cotransporter-2 (SGLT2) inhibitors that can provide cardiorenal protection.^13–15^

Although CKD is well-defined using eGFR and UACR, the rate of diagnosis coding of CKD remains poor within UK primary care,^16,17^ despite existing financial incentives that promote identification and management of CKD under the Quality and Outcomes Framework (QOF). Yet, diagnostic coding of CKD in primary care is associated with lower rates of hospitalisation for cardiovascular events,^18^ and improved quality of care.^17^ Existing work exploring the rate of CKD diagnosis coding in UK primary care has focused on the general population, with diabetes considered as a subgroup.^16,17^ However, people with T2D engage with primary care services differently than the general population. It is therefore important to quantify how well CKD is coded in a population with T2D, within the context of the clinical guidelines relevant to this group.

This study aims to establish the incidence of CKD estimated using eGFR-based evidence and/or diagnosis codes, the proportion of eGFR-based CKD incidences that have a diagnosis code, and the timeliness of CKD diagnosis coding in a cohort of patients with T2D.

## Method

This is a retrospective cohort study using routinely collected UK primary care data from 60 general practices across England between February 2012 and December 2022.

### Cohort

All patients with T2D were considered for inclusion in the study cohort and considered at risk of incident CKD, regardless of whether their serum creatinine was measured throughout follow-up, providing an intention-to-treat population estimate. CKD incidence estimates were therefore not impacted by non-adherence to clinical guidelines for the monitoring and diagnosis of kidney disease.

A burn-in period was defined from the start of data collection (February 2012) to 5 April 2015 to exclude patients with pre-existing CKD (either eGFR-based or coded) before the study index date (6 April 2015); any patients with evidence of CKD within the burn-in period or before their diagnosis of T2D were excluded. The study period ran from the 2015–2016 to 2020–2021 fiscal years.

### Definitions

eGFR-based CKD was ascertained using repeated serum creatinine or eGFR measurements, with eGFR calculated from serum creatinine using the 2021 Chronic Kidney Disease Epidemiology Collaboration (CKD-EPI) formula^19^ without racial adjustment. If both an eGFR and serum creatinine measurement existed on the same day for a patient, the serum creatinine measurement was retained and used to calculate eGFR. Patients were classed as having eGFR-based CKD if they had two or more eGFR measurements <60 ml/min/1.73 m^2^ at least 90 days apart, up to a maximum of 15 months apart. Any eGFR measurements occurring between the dates of those observations were required to have a median of <60 ml/min/1.73 m^2^ .

An upper limit on the time between qualifying eGFR measurements was imposed to distinguish between a sustained drop in kidney function and two acute episodes of kidney dysfunction. This upper limit was set to 15 months between measures to allow identification of eGFR-based CKD using measurements obtained at two consecutive diabetic annual reviews.

Diagnosis codes were identified using Read and SNOMED terminologies. CKD diagnoses of stages G3–5 (eGFR <60) were classed as CKD within this analysis. Codelists are provided in Supplementary Tables S4, S5, and S6.

### Statistical analysis

The number of patients with a diagnosis code and/or meeting the eGFR-based criteria for CKD were estimated for each included fiscal year, to align with the financial incentives and audits within the NHS. We further present the number (and percentage) of patients who have at least one observation of eGFR (or serum creatinine) during that year, and how many of these have at least one eGFR <60 ml/min/1.73 m^2^ .

To estimate the annual incidence of CKD, three at-risk cohorts of patients were established (one for each definition of CKD) for each fiscal year from 2015–2016 to 2020–2021. Patients are considered at-risk if they are registered with a participating GP practice with a diagnosis of T2D before the end of the fiscal year, and no prior evidence of CKD. Evidence of CKD was defined as having:

1. eGFR-based CKD;
2. a CKD diagnosis code;
3. eGFR-based CKD or a CKD diagnosis code (composite criteria).

Incidence rates are presented per 100 person–years along with Poisson-based 95% confidence intervals (CIs). Follow-up began at the latest of the index date, or date of T2D diagnosis. Follow-up ended at the earliest of death, CKD incidence, GP practice deregistration, or the end of the study period.

We performed a sensitivity analysis on the eGFR formula, using the CKD-EPI 2009^20^ and Modification of Diet in Renal Disease (MDRD)^21^ formulae. We described the changes in the eGFR estimates and their impact on CKD incidence rates.

For eGFR-based CKD, we estimated the median time interval between the two qualifying eGFR measurements. We quantified the number and proportion of patients with eGFR-based CKD that also received a CKD diagnosis code, and summarised the attributes of patients with and without CKD diagnosis codes descriptively, using the median and interquartile range for continuous measures, and count and percentage for categorical measures: age, sex, deprivation, duration of diabetes, eGFR-stage, time between qualifying eGFRs, UACR, HbA1c control, cardiovascular disease, and indicators of medication prescribing. UACR, HbA1c, and prescribing indicators were extracted from the year before first qualifying eGFR. Logistic regression was used to identify which patient attributes were associated with entry of a CKD diagnosis code. Sex, eGFR, UACR, deprivation decile, HbA1c control, cardiovascular disease, and medication indicators were included as categorical predictors, and age, duration of diabetes, and time between qualifying eGFRs were included as continuous predictors. Deprivation decile is assigned using the postcode of a patient’s GP practice, from 1 = most deprived to 10 = least deprived.

For patients who met the eGFR-based criteria and had a CKD diagnosis code during follow-up, we quantified the number and proportion of patients who received a diagnosis code before and after the second qualifying eGFR observation. When a patient met the eGFR-based criteria and had a CKD diagnosis code after their second qualifying measurement, we defined this as clinician-verified CKD. The proportion of patients with eGFR-based CKD that had clinician-verified CKD was estimated and the time to verification was summarised using the median and interquartile range.

All analyses were conducted in R (version 4.2.3).

## Results

**Figure 1.**
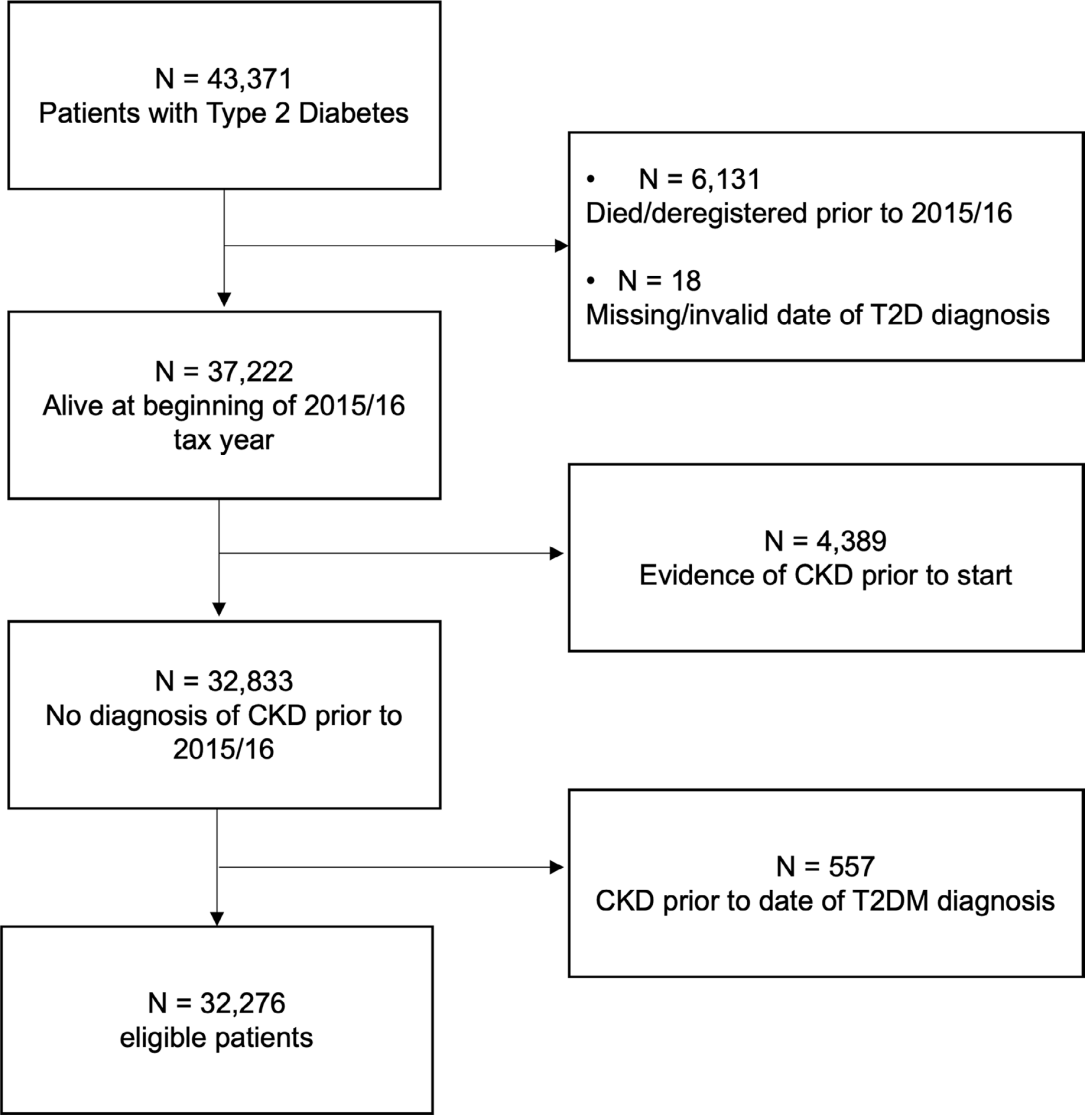
Flowchart of patient inclusions and exclusions

A total of 32 276 patients had T2D and were at risk of CKD (Figure 1). Of these, 30 081 (93.2%) had at least one eGFR or serum creatinine measurement recorded during follow-up. In total, 13 945 patients (43.2%) were receiving care from a GP practice in a highly deprived area (Index of Multiple Deprivation [IMD] decile = 1 or 2) and the majority of patients had been first diagnosed with T2D in the 2 years before study entry (median 1.8 years; IQR 0–7 years), with an average follow-up of 5 years (IQR: 2.31, 6.00) (Table 1).

**Table 1.** Characteristics of patient cohort at the time of cohort entry

Variable	Patients with T2D and no previous evidence of CKD stages G3–5 (*n* = 32 276)
Median age, years (IQR)	60 (50–70)
Sex, *n* (%)	
Female	13 865 (43.0)
Male	18 411 (57.0)
Median duration of follow-up, years (IQR)	5.01 (2.31–6.00)
Median duration of diabetes, years (IQR)	1.8 (0.0–7.0)
CKD diagnosis code, *n* (%)	1463 (4.5)
Median time to diagnosis code, years (IQR)	3.10 (1.49–5.41)
eGFR-based CKD, *n* (%)	2667 (8.3)
Median time to lab-based CKD, years (IQR)	2.66 (1.26–4.41)
Lab-based or coded CKD, *n* (%)	3102 (9.6)
Median time to lab-based or coded CKD, years (IQR)	2.52 (1.12–4.36)
Deprivation decile, *n* (%)	
1–2 (highly deprived)	13 945 (43.2)
3–4	6070 (18.8)
5–6	3309 (10.3)
7–10 (least deprived)	8952 (27.7)
General practices*, n*	60
Median number of patients per practice (IQR)	472 (277–603)

CKD = chronic kidney disease. eGFR = estimated glomerular filtration rate. IQR = interquartile range. T2D = type 2 diabetes.

At least one measurement of eGFR <60 ml/min/1.73 m^2^ was observed in 4351 patients. However, only 3000 (68.9%) had a follow-up eGFR (serum creatinine) within 6 months of their first abnormal result (Supplementary Figure S3).

### CKD incidence

**Figure 2.**
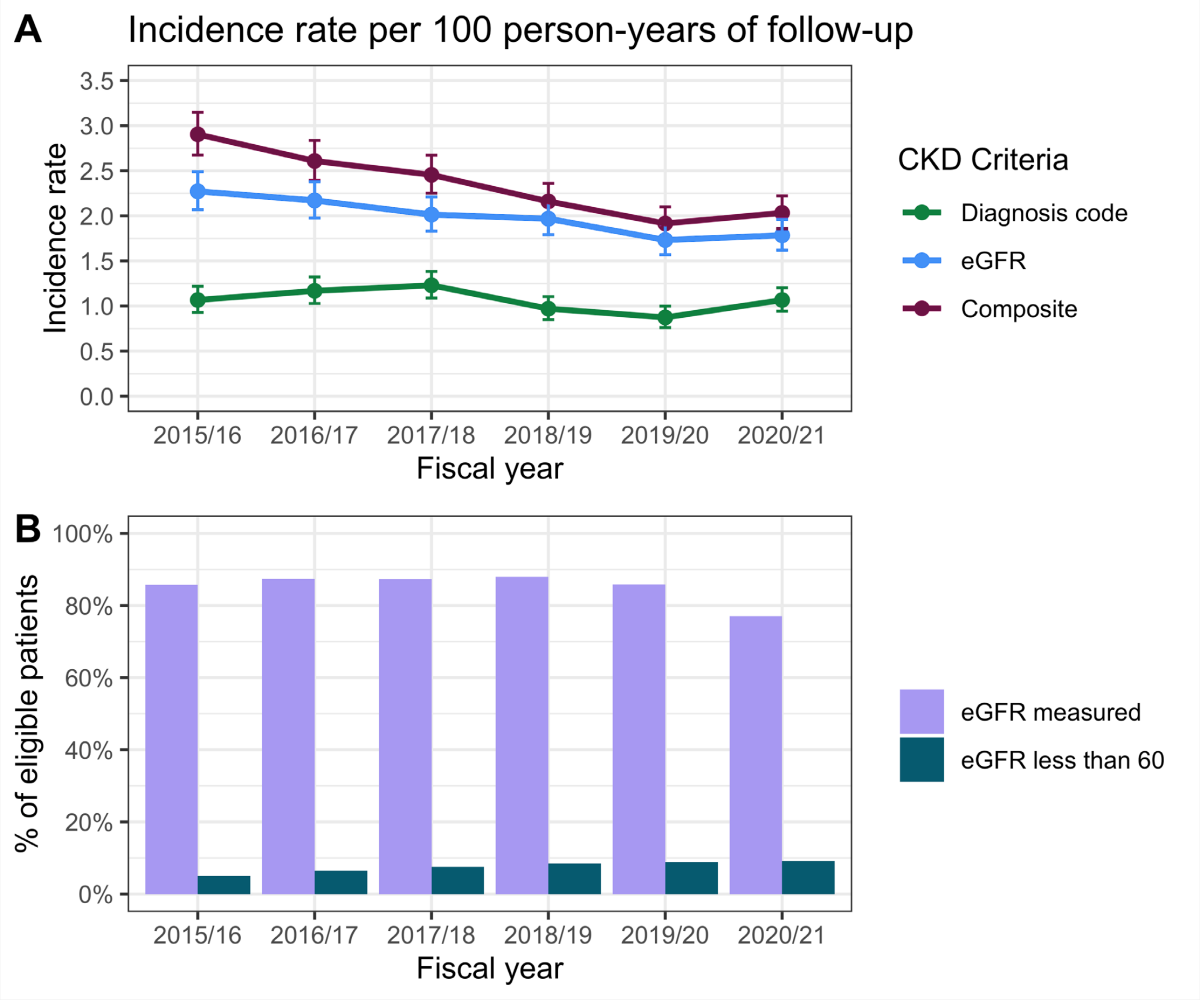
Among people with type 2 diabetes and no previous evidence of CKD stage G3–5, (**A**) The incidence rates of CKD diagnosis via a diagnosis code (code), eGFR-based criteria (lab) or at least one of these (either), and their 95% confidence intervals. (**B**) Rates of eGFR measurement, by fiscal year. CKD = chronic kidney disease. eGFR = estimated glomerular filtration rate.

Over the study period, the incidence rate of CKD was 2.32 (95% CI = 2.24 to 2.41) per 100 person–years, using the composite definition of eGFR-based criteria or presence of a CKD diagnosis code. The CKD incidence estimates using eGFR-based criteria only and diagnosis codes only were 1.98 (95% CI = 1.90 to 2.05) and 1.06 (95% CI = 1.00 to 1.11), respectively. The combined incidence estimate across all fiscal years was significantly higher in the composite criteria than either the eGFR-based criteria (*P*<0.001) or diagnosis code criteria (*P*<0.001). Incidence estimates using only CKD diagnosis codes significantly underestimated the overall incidence, as estimated using the composite criteria, across all annual estimates. Incidence estimates using only the eGFR-based criteria were not significantly different from the composite criteria in fiscal years 2018–2019, 2019–2020 and 2020–2021 (Figure 2A, Supplementary Table S1).

The incidence rate of CKD using eGFR-based criteria was higher when estimated using the CKD-EPI 2009 formula (2.36 [95% CI = 2.28 to 2.44]) or Modification of Diet in Renal Disease (MDRD) formula (2.44 [95% CI = 2.36 to 2.53]) compared with the CKD-epi 2021 formula.

Approximately, 1-in-4 diagnosis code cases did not have evidence of eGFR-based CKD (*n* = 411, 28.1%). Whereas, more than 1-in-2 eGFR-based cases did not have a corresponding diagnosis code (*n* = 1457, 54.6%). These findings were consistent over time (Supplementary Table S1). Using either the CKD-EPI 2009 or MDRD formulae to estimate eGFR resulted in a higher proportion of eGFR-based cases without a corresponding diagnosis code (59.2% and 61.7%, respectively).

There was a statistically significant drop (*P*<0.001) in the proportion of patients receiving at least one eGFR (serum creatinine) measurement in the 2020–2021 fiscal year to 77.0% (95% CI = 76.5% to 77.5%), from 85.8% (95% CI = 85.4% to 86.2%) in 2019–2020. Before 2019–2020, the proportion of eligible patients with at least one valid eGFR or serum creatinine measurement remained more than 80% (Figure 2B). The proportion of patients with an eGFR <60 ml/min/1.73 m^2^ increased over time from 5.1% (95% CI = 4.8% to 5.4%) in 2015–2016 to 9.1% (95% CI = 8.7% to 9.4%) in 2020–2021 (Figure 2B).

### Diagnostic coding of patients meeting eGFR-based criteria

In total, 2667 patients (8.3%) met the eGFR-based criteria for CKD during the study period. Of these, 54.6% patients did not have a corresponding diagnostic code during follow-up (Supplementary Table S2).

Patients who had eGFR-based evidence of CKD were more likely to have a diagnosis code if they were younger, had a CKD stage G4 at the first qualifying eGFR observation, an observed UACR (stage A1) or no observed HbA1c in the past year (Table 2).

**Table 2.** Odds ratios (OR) and 95% confidence intervals from a logistic regression model to identify factors associated with diagnostic coding of eGFR-based cases

Variable	Group	OR (95% CI)	*P* value
Age		0.973 (0.963 to 0.982)	0.00
Sex	Female (ref)		
	Male	1.069 (0.913, to1.252)	0.41
Duration of diabetes, years		0.999 (0.991 to 1.008)	0.89
Deprivation decile	1–2 (ref)		
	3–4	1.090 (0.945 to 1.257)	0.24
	5–6	1.028 (0.865 to 1.222)	0.75
	7–10	0.942 (0.773 to 1.148)	0.55
eGFR stage	G3A (ref)		
	G3B	1.206 (0.932 to 1.559)	0.15
	G4	1.796 (1.074 to 3.046)	0.03
	G5	1.023 (0.365 to 2.859)	0.97
Time between qualifying eGFRs, months		0.853 (0.647 to 1.124)	0.26
Urine albumin-to-creatinine ratio	Not measured (ref)		
	A1	1.298 (1.081 to 1.559)	0.01
	A2	1.078 (0.855 to 1.360)	0.52
	A3	1.310 (0.904 to 1.904)	0.15
HbA1c control	Controlled (ref)		
	Uncontrolled	1.105 (0.920 to 1.329)	0.29
	Unmeasured	1.502 (1.163 to 1.941)	0.00
History of cardiovascular disease		1.080 (0.878 to 1.329)	0.46
Prescribed antihypertensives		0.980 (0.795 to 1.210)	0.85
Prescribed statins		0.995 (0.845 to 1.172)	0.95
Prescribed insulin		1.194 (0.965 to 1.478)	0.10
Prescribed SGLT2 inhibitors		0.904 (0.563, to 1.447)	0.67

eGFR = estimated glomerular filtration rate. SGLT2 = sodium-glucose co-transporter-2.

### Timeliness of diagnosis coding

The majority of patients (55.2%) with both eGFR-based CKD and a CKD diagnosis code received their diagnosis code after their second qualifying eGFR measurement (Table 3), and the median time from the second qualifying eGFR to entry of a diagnosis code was 9.8 months (IQR: 1.2, 24.3). Meanwhile, 23.8% of patients with clinician-verified CKD received a diagnosis code within 30 days of meeting the eGFR-based criteria, 31.6% within 90 days, and 40.1% and 56.1% within 6 and 12 months, respectively.

**Table 3.** Number of patients with eGFR-based chronic kidney disease (CKD) and a CKD diagnosis code, the proportion that occurred on or after their second qualifying eGFR measurement and the time between the second qualifying eGFR measurement and entry of a diagnostic code

Variable	Group	Patients, *n*	Diagnosis code after eGFR-based CKD, *n* (%)	Median time from eGFR-based CKD to CKD diagnosis code, months (IQR)
Age group, years				
	<40	3	1 (33.3)	0.7 (0.7–0.7)
	40–49	19	11 (57.9)	2.9 (1.9–9.6)
	50–59	111	56 (50.5)	8.9 (1.1–16.3)
	60–69	298	156 (52.3)	10.9 (2.0–28.2)
	70–79	496	293 (59.1)	9.9 (1.0–24.8)
	≥80	283	151 (53.4)	8.9 (0.9–20.6)
Sex				
	Female	566	319 (56.4)	10.6 (1.1–24.1)
	Male	644	349 (54.2)	9.0 (1.2–24.3)
Duration of T2DM				
	<1 year	77	48 (62.3)	11.8 (0.8–26.2)
	1–2 years	69	44 (63.8)	10.1 (2.0–27.2)
	2–5 years	177	94 (53.1)	7.6 (0.7–26.1)
	5–10 years	359	179 (49.9)	9.7 (1.1–21.4)
	>10 years	528	303 (57.4)	10.2 (1.5–23.2)
CKD stage				
	G3A	1025	569 (55.5)	9.9 (1.2–25.3)
	G3B	139	78 (56.1)	10.2 (1.2–20.4)
	G4	38	17 (44.7)	7.3 (0.7–16.6)
	G5	8	4 (50.0)	7.2 (2.8–21.5)
Total	Overall	1210	668 (55.2)	9.8 (1.2–24.3)

eGFR = estimated glomerular filtration rate. CKD = chronic kidney disease. IQR = interuartile range. T2DM = type 2 diabetes mellitus.

In total, 469 patients had at least one additional eGFR measurement between their CKD-qualifying eGFR<60 and entry of a CKD diagnosis code. At least one measurement of an eGFR <60 ml/min/1.73 m^2^ was observed in 4351 patients. However, only 3000 (68.9%) had a follow-up eGFR (or serum creatinine) within 6 months of their first abnormal result (Supplementary Table S3, Supplementary Figure S1). Ninety-four patients had an eGFR reported on the same date as their diagnosis code, and these were on average 2.36 ml/min/1.73 m^2^ lower than their CKD-qualifying eGFR (Supplementary Figure S3), *P* = 0.019.

## Discussion

### Summary

We have provided concerning evidence of a lack of diagnosis coding of CKD in patients with T2D in UK primary care, with more than half of patients meeting the eGFR-based CKD criteria never receiving a diagnosis code. Of those patients who have a CKD diagnosis code after meeting the eGFR-based criteria, diagnosis coding occurs with a median delay of more than 9 months. CKD incidence was severely underestimated when using CKD diagnosis codes alone, which prevents reliable quantification of the epidemiology and burden of CKD.

### Strengths and limitations

To the best of our knowledge, this is the first study to quantify the rates of CKD diagnosis coding for new incidences of CKD in a population with T2D in UK primary care. We provide an analysis of recent data that covers the onset of the COVID-19 pandemic and therefore any associated impact on the management and identification of CKD. We hypothesise that the observed drop in eGFR measurement rates can be attributed to disruptions to the provision of routine care caused by the COVID-19 pandemic.^22^ A limitation to this work is that our data only cover primary care. Patients may be referred to and managed within specialist secondary care centres on exhibition of kidney function impairment. However, these referrals should be evidenced within the patient’s primary care record. Future work should explore how patients diagnosed with CKD in primary care are managed across the care pathway.

A further limitation is that our lab-based CKD definition used only eGFR. Albuminuria and/or an elevated UACR is often an earlier signal for kidney damage than reduced eGFR and is common in patients with T2D. Therefore, further work should also capture patients that present with persistent albuminuria. However, adherence to UACR measurement guidelines is poor, so incidence estimates based on UACR derived using routinely collected data are likely to be unreliable.

A final limitation is that there is considerable variability in how eGFR is estimated by each studied formula (eGFR CKD-EPI 2009 and 2021 and MDRD). This raises doubt concerning the reliability and precision of such formulae; however, we have used CKD-EPI 2021 which provides the most conservative (that is, highest) estimates of eGFR. We have therefore mitigated the risk of overdiagnosis of CKD based on eGFR by applying the formula that comparatively detects fewer cases of CKD.

### Comparison with existing literature

Similarly to González-Pérez *et al*,^23^ we have presented crude estimates of CKD incidence per 100 person–years by ascertainment criteria (eGFR-based CKD, diagnosis coded CKD, or either). However, our combined estimates were considerably lower, likely owing to several differences in our criteria definitions; our study does not identify cases using UACR or albuminuria measurements, which accounted for 49% of González-Pérez *et al*’s included incidences. Furthermore, their work did not impose an upper limit on the time between qualifying eGFR measurements.

Others have reported higher estimates of the proportion of lab-based CKD cases with a corresponding diagnosis code ranging from 57.5%–77.7% for patients with diabetes in UK primary care.^16,17^ However, these studies covered an earlier timeframe, focused on prevalence rather than incidence, and defined eGFR-based CKD differently to the present study.

### Implications for research and practice

A lack of, or delay in, identification and diagnosis coding of CKD could lead to improper management of the condition. CKD progression is strongly associated with poor clinical outcomes and has a significant economic burden. CKD awareness remains profoundly low, in part because CKD is usually silent until its late stages. Physician awareness of CKD is critical in the early identification of the condition as well as the early implementation of evidence-based therapies that can slow progression of kidney dysfunction, prevent metabolic complications, and reduce cardiovascular-related outcomes. Tools, including automated CKD patient registry and diagnostic coding, within electronic health records to identify and prioritise patients for early intensive management, can facilitate the clinical inertia we see in CKD management.^24^

In conclusion, this study has provided evidence that CKD is poorly coded in primary care for people with T2D, despite annual kidney function testing, which could lead to improper care and delayed intervention. The reasons for poor coding require further investigation, and emphasis should be placed on examining historic test results for CKD to improve diagnosis coding.

## Data

It is not appropriate to publish the research dataset associated with this work since it contains individual-level NHS-held patient data that cannot be shared beyond the approved research team.
